# Adaptive neural network projection analytical fault-tolerant control of underwater salvage robot with event trigger

**DOI:** 10.3389/fnbot.2022.1082251

**Published:** 2023-02-02

**Authors:** Gaoyang Guo, Qiang Zhang, Yan Zhang, Wenyi Tan, Zewen Tao, Sainan Ma

**Affiliations:** ^1^Shandong Key Laboratory of Intelligent Transportation (Preparatory), School of Navigation and Shipping, Shandong Jiaotong University, Weihai, China; ^2^Shandong Future Robot Co., Ltd., Weihai, China; ^3^Zhejiang Jialan Ocean Electronics Co., Ltd., Zhoushan, China

**Keywords:** over-drive fault-tolerant control, thruster failure, projection analysis, underwater salvage robot, adaptive neural network

## Abstract

**Introduction:**

To solve the problem of control failure caused by system failure of deep-water salvage equipment under severe sea conditions, an event-triggered fault-tolerant control method (PEFC) based on proportional logarithmic projection analysis is proposed innovatively.

**Methods:**

First, taking the claw-type underwater salvage robot as the research object, amore universal thruster fault model was established to describe the fault state of equipment failure, interruption, stuck, and poor contact. Second, the controller was designed by the proportional logarithmic projection analytical method. The system input signal was amplified and projected as a virtual input, which replaces the original input to isolate and learn the fault factor online by the analytical algorithm. The terminal sliding mode observer was used to compensate for the external disturbance of the system, and the adaptive neural network was used to fit the dynamic uncertainty of the system. The system input was introduced into the event-triggered mechanism to reduce the output regulation frequency of the fault thruster.

**Results:**

Finally, the simulation results showed that the method adopted in this study reduced the power output by 28.95% and the update frequency of power output by 75% compared with the traditional adaptive overdrive fault-tolerant control (AOFC) method and realized accurate pose tracking under external disturbance and system dynamic uncertain disturbance.

**Discussion:**

It has been proven that the algorithm used in this research can still reasonably allocate power to reduce the load of a fault thruster and complete the tracking task under fault conditions.

## 1. Introduction

**Figure 1:**
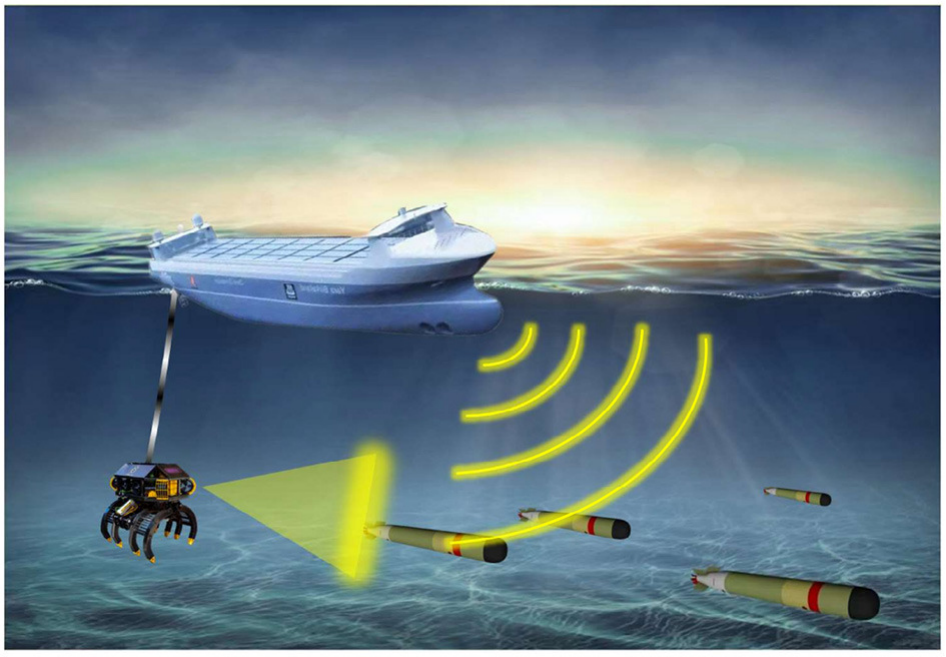
A schematic diagram of a claw underwater salvage robot.

The twenty-first century is the century of the ocean. With the gradual deepening of the development of marine resources in various countries, an increasing number of maritime accidents have led to the loss of a large number of high-value property in the sea. The traditional automatic salvage equipment is designed for the separation of salvage fixtures and underwater search vehicles, and the salvage steps have a low success rate. The underwater salvage robot studied in this article is a claw-type underwater salvage robot, which mainly grabs underwater cylindrical salvage objects, such as training torpedo bomb with failure of a floating system, ship wreck column structure, and so on. In practical engineering application, the mother ship carries the robot sailing to the target water area, the diving depth of the robot is controlled by the armored steel cable after the lifting system is lowered to the target depth, and the underwater search is carried out by following the mother ship through its own power. The robot accurately locates the position of the salvage. Finally, the attitude adjustment thruster is turned on to adjust the relative position and use the lower claw of the robot to grab the salvage object, which is then recovered by the mother ship as shown in Figure 1. Underwater salvage robots working in complex and harsh sea conditions for a long time will face the following challenges in control design: 1) When the control process is affected by modeling technology, environmental uncertainty and parameter uncertainty, how to realize the high accuracy and high sensitivity control of underwater vehicle under complex sea conditions; 2) The fault-tolerant control effect of the thruster of the underwater vehicle under the condition of unknown faults such as line damage, foreign body damage, and so on; 3) In the case of lack of power in the fault state, the high-frequency control system and high-load transfer propulsion system leading to accelerated wear damage of the fault thruster; and 4) the isolation adaptation of multiplicative coupling faults and the accurate compensation of the controller.

For actuator fault-tolerant control, there are passive fault-tolerant control (Jin, 2016) and active fault-tolerant control (Zhao et al., 2018). The design principle of passive fault-tolerant control is to enhance the robust performance of the controller and improve the control effect of equipment in the presence of external disturbances and internal faults, but the strong robustness of passive fault-tolerant control is worse than that of active fault-tolerant control. At present, the mainstream solution to equipment actuator failure is active fault-tolerant control, in which many scholars only study one of the cases: either invalid fault or stuck fault. Thruster invalid failure is due to oil leakage in the hydraulic system or sea water corrosion and other force majeure factors caused by part of the propeller blade damage, resulting in much less the actual output power than the expected power. This kind of fault is common in practical engineering, but it is difficult to control the deep coupling between the fault and the system model. Most of them improve system robustness by magnifying parameter excitation persistence or the excitation threshold (Chen et al., 2018) and design robust fault observers to increase the accuracy of system compensation and improve system robustness (Park and Yoo, 2016). The reason for the stuck fault is that the propulsion system cannot effectively control the pitch of the propeller caused by the suction of foreign bodies such as underwater suspended plants or the control system loses control of the thruster due to the failure signal of the transmission line controlled by the fuselage. The propulsion system maintains the final thrust output of effective control, which will make some of the thrusters completely out of control and cause strong interference to the system as a whole. In order to solve the problem of stuck faults caused by communication faults, the residual system constructed by the model is approximated to enhance the robustness of the system to faults and external disturbances (Yan and Ren, 2021). In order to improve the control effect of the system fault, Zong et al. (2020) extended the system fault to the system state quantity and designed an observer to approximate the system fault state and external disturbance state. Van et al. (2016) have designed an active fault-tolerant control algorithm for the non-singular terminal sliding surface to solve the external unknown disturbance and system jam fault. The previously mentioned five control algorithms mainly solve the thruster failure or paranoid jamming in the system, but the fault situation in the actual engineering control is often unpredictable, which makes the application environment considered by the algorithm design is not comprehensive.

To improve the ability of the controller to deal with multiple faults, the solution presents two schools: 1) establish a more complete fault model and 2) add a fault detection module to assist control decision-making. A more perfect dynamic model of fault description is established, and then adaptive robust control such as adaptive sliding mode controller (Hao et al., 2020) is designed to estimate fault information and unknown disturbance upper bound of the system (Zhu et al., 2021). The engineering problem of complex fault of actuator in bad sea condition is solved, but the control phenomenon of deep coupling between actuator fault and dynamic uncertainty of system model occurs. The design idea of this school is to design fault-tolerant controller directly for coupling parameters. However, there will be the problem that fault-tolerant control compensation is not sensitive and is easily affected by model dynamics. In order to solve the problem of fault tolerance in the process of ship motion, Benetazzo et al. (2015) used the parity space method and Luenberger observer to detect the fault of the system. In order to further improve the control accuracy, Kalman filter (Cristofaro and Johansen, 2014) interference compensation is designed on the basis of the controller for fault-tolerant control of the overactuated marine vessel. When there is a fault of the actuator, a new dynamic state inevitably appears. It is a processing idea to reconstruct the power (Yang et al., 1999; Liu et al., 2022) of the actuator after fault detection and carry out fault-tolerant control. The earlier design scheme needs to rely on the feedback of the system fault detection module to the system control, but in the actual project, the complex fault situation, environmental noise, and the particularity of the system structure will lead to misreport and underreport. It will directly affect the accuracy and sensitivity of the fault-tolerant controller.

The earlier control schemes only consider the fault-tolerant control for actuator faults and seldom consider that the load capacity of the propulsion device of the system decreases after the actuator failure, and the high-frequency regulation load will lead to further damage to the propulsion device. This makes the equipment out of control without the knowledge of the operator. Now the event trigger mechanism has a great advantage in reducing the signal transmission frequency and reducing the thruster adjustment times. Among them, the trigger environment of the preset event (Tabuada, 2007; Xu et al., 2018; Wang et al., 2019) is more common, which makes the system feedback enter the preset event trigger evaluation condition and input the actuator, and the trigger effect is also highly dependent on the preset event trigger condition. Therefore, the design of dynamic event trigger mechanism which adjusts with time and system state has become the key factor to effectively reduce the load of system regulation. Event triggering mechanism has been effectively applied to trajectory tracking (Deng et al., 2019b; Chen et al., 2022), path tracking (Li et al., 2020), and formation control of surface and underwater vehicles.

**Figure 2:**
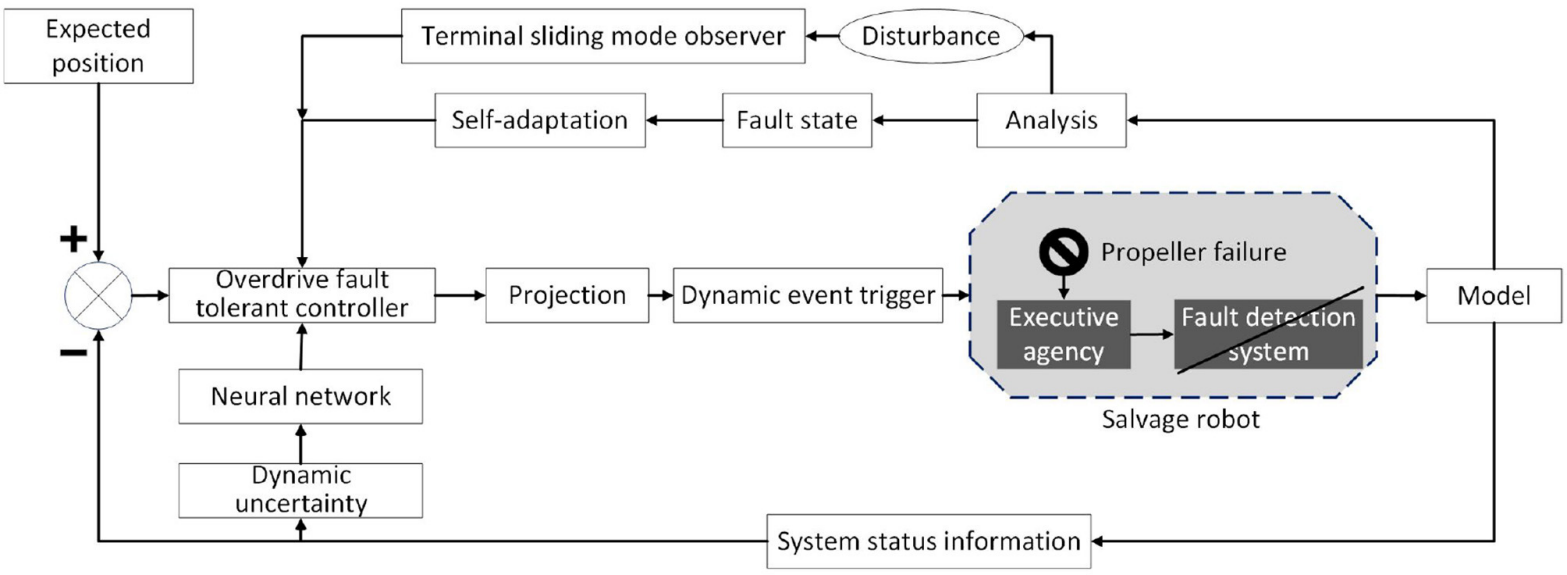
Schematic diagram of a fault-tolerant control scheme for proportional logarithmic projection analysis of overdrive.

In order to solve the earlier problems, this article first designs and improves a more universal fault model of the underwater salvage robot, and designs a proportional logarithmic projection analytical overdrive trigger controller according to the assembly features of the robot propulsion system to control the dynamics of the outer loop. The separation characteristic of the projection analytical controller is used to isolate and adapt the deeply coupled fault feature state. By making the control system of the underwater salvage robot get rid of the assistance of the fault detection system and the monitoring and sensing system of the thruster as shown in Figure 2, it can still achieve accurate fault compensation and reasonable distribution of power to reduce the output of the fault thruster. The terminal sliding mode observer is used to observe and compensate the bounded disturbance of the external ocean current and the paranoid fault disturbance of the thruster in real-time, and the adaptive extension network is used to fit the dynamic uncertainty of the system online to improve the accuracy and sensitivity of the system. In order to solve the problem of frequent adjustment and high output after system thruster failure, the dynamic event trigger mechanism is introduced. Finally, it is proved that the controller is bounded and convergent in trajectory tracking control by Lyapunov stability analysis (Deng et al., 2019a).

## 2. Construction of motion model of underwater salvage robot

According to the motion characteristics of the underwater salvage robot, the motion of the surge, sway, and yaw of the underwater robot is considered to be decoupled, and the water surface control ship controls the active water depth of the underwater robot by releasing the cable length, so the underwater robot heave, roll, and pitch motion dimensions are ignored. The motion model of the underwater salvage robot (Corradini et al., 2010) is simplified to a three-degree-of-freedom underwater motion model in the horizontal longitudinal-transverse plane according to the working environment and design features. The surge, sway, and yaw of the underwater salvage robot are controllable variables of the control system. In the reference article Fossen (2011), the position vectors of the underwater vehicle coordinate system and the geodetic coordinate system are [*x, y*, ψ]^T^. Under geodetic coordinates, the position coordinates of the underwater vehicle are (*x, y*). The velocity vector is [*u, v, r*]^T^. Therefore, the motion model of this underwater salvage robot is described as follows:


(1) $$\begin{array}{l} \{\begin{array}{l} \dot{x}=u\cos\psi -v\sin\psi \\ \dot{y}=u\sin\psi +v\cos\psi \\ \dot{\psi }=r\\ \dot{u}=\frac{1}{m_{11}}\tau_{\text{u}}+\frac{1}{m_{11}}\tau_{\text{wu}}-\frac{F_{u}(x)}{m_{11}}\\ \dot{v}=\frac{1}{m_{22}}\tau_{\text{v}}+\frac{1}{m_{22}}\tau_{\text{wv}}-\frac{F_{v}(x)}{m_{22}}\\ \dot{r}=\frac{1}{m_{33}}\tau_{\text{r}}+\frac{1}{m_{33}}\tau_{\text{wr}}-\frac{F_{r}(x)}{m_{33}} \end{array} \end{array}$$

In the equation $m_{11}=m-X_{\dot{u}}$, $m_{22}=m-Y_{\dot{v}}$, *m*_33_ = *I*_*z*_ − *N*_ṙ_, $F_{u}\left(x\right)=-\left(m-Y_{\dot{v}}\right)vr-\left(X_{u}+X_{u|u|}|u|\right)u$, $F_{v}\left(x\right)=\left(m-X_{\dot{u}}\right)ur-\left(Y_{v}+Y_{v|v|}|v|\right)v$, $F_{r}\left(x\right)=\left(X_{\dot{u}}-Y_{\dot{v}}\right)uv-\left(N_{r}+N_{r|r|}|r|\right)r$, *m* is the underwater weight for underwater salvage robot, *I*_z_ is the moment of inertia for underwater salvage robot, *X*_u˙_, *Y*_v˙_, and *N*_ṙ_ are the additional mass of the underwater salvage robot in the three dimensions of advance, drift, and yaw, respectively. τ_*u*_, τ_*v*_, and τ_*r*_ are control force and torque of the underwater salvage robot in the three dimensions of advance, drift, and yaw, respectively. τ_wu_, τ_wv_, and τ_wr_ are the unknown bounded interference force and torque produced by the horizontal flow and vertical flow of the underwater ocean current in the three dimensions of advance, drift, and yaw, respectively. *X*_*u*_, *Y*_*v*_, and *N*_*r*_ are the linear damping terms of the underwater salvage robot in the three dimensions of advance, drift, and yaw, respectively. *X*_*u*|*u*|_|*u*|, *Y*_*v*|*v*|_|*v*|, and *N*_*r*|*r*|_|*r*| are the nonlinear damping terms of the underwater salvage robot in the three dimensions of advance, drift, and yaw, respectively.

## 3. Design of motion inner loop controller

A fault-tolerant controller is designed because the thrust system of the system is prone to thruster failure, interruption, jam, and poor contact when working for a long time in a deep and high-pressure environment, so the controller needs to compensate for the output of the equipment thruster. To realize the direct control of force and torque, the controller is designed as the inner loop control.

Design kinematic and dynamic error formulas for surge, sway, and yaw directions.


(2) $$\begin{array}{l} \{\begin{array}{l} e_{1}=x-x_{\text{d}}\\ u_{\text{e}}=u-u_{\text{d}}\\ e_{2}=y-y_{\text{d}}\\ v_{\text{e}}=v-v_{\text{d}}\\ e_{3}=\psi -\psi_{\text{d}}\\ r_{\text{e}}=r-r_{\text{d}} \end{array} \end{array}$$

The kinematics Lyapunov functions of surge, sway, and yaw direction are designed to solve the virtual kinematics control law.


(3) $$\begin{array}{l} V=\frac{1}{2}{e_{1}}^{2}+\frac{1}{2}{e_{2}}^{2}+\frac{1}{2}{e_{3}}^{2} \end{array}$$

By taking the derivative of Equation (3) and substituting (Equations 1, 2):


(4) $$\begin{array}{l} \dot{V}=e_{1}(u_{\text{e}}\cos\psi +u_{\text{d}}\cos\psi -v\sin\psi -{\dot{x}}_{\text{d}})+e_{2}(u\sin\psi \\ +v_{\text{e}}\cos\psi +v_{\text{d}}\cos\psi -{\dot{y}}_{\text{d}})+e_{3}(r_{\text{e}}+r_{\text{d}}-{\dot{\psi }}_{\text{d}}) \end{array}$$

According to Equation (4), the inner loop desired velocity *u*_d_, *v*_d_, and *r*_d_ are designed as


(5) $$\begin{array}{l} u_{\text{d}}=\frac{1}{\cos\psi }\left(v\sin\psi +{ẋ}_{\text{d}}-k_{1}e_{1}\right) \end{array}$$

(6) $$\begin{array}{l} v_{\text{d}}=\frac{1}{\cos\psi }\left({ẏ}_{\text{d}}-u\sin\psi -k_{3}e_{2}\right) \end{array}$$

(7) $$\begin{array}{l} r_{\text{d}}={\dot{\psi }}_{\text{d}}-k_{5}e_{3} \end{array}$$

Substituting (Equations 5–7) into Equation (4):


(8) $$\begin{array}{l} \dot{V}=-k_{1}{e_{1}}^{2}-k_{3}{e_{2}}^{2}-k_{5}{e_{3}}^{2}+{Y_{\text{e}}}^{\text{T}}u_{\text{p}} \end{array}$$

in the equation $u_{p}={\left[e_{1}\cos\psi ,e_{2}\cos\psi ,e_{3}\right]}^{\text{T}}$,${Y_{e}}^{\text{T}}=\left[u_{\text{e}},v_{\text{e}},r_{\text{e}}\right]$.

## 4. Dynamic outer loop controller design

Taking the virtual input of the inner loop controller as the speed expectation of the outer loop dynamic control, the actual torque input of the system is controlled by the Backstepping design idea. Since the multi-drive motion fault is considered in this article, an overdrive dynamics controller is designed.

**Lemma 1:** Composite disturbance observer (Equations 23, 24) uses $\dot{\Delta }$, which can be obtained in a finite time using a first-order sliding mode differentiator. For the specific form, please refer to the reference (Levant, 2003).

**Lemma 2:** According to system (Equation 1), in *n* input *n* output system, Radial Basis Function (RBF) (Chen et al., 2022) has the ability to approximate nonlinear terms, $Z_{n}\in R^{n}$ compact emergency can get


(9) $$\begin{array}{l} F_{n}\left(x\right)=W_{n}^{*\text{T}}\psi_{n}\left(z_{n}\right)+\varepsilon , \end{array}$$

Where ε is the approximation error, $W_{n}^{*\text{T}}$ is the weight matrix, ψ_*n*_(*z*_*n*_) is the activation function vector. $\bar{\varepsilon }$ is the boundary of ‖ε‖, and $\sup_{z_{n}\in R^{n}}\|F_{n}\left(x\right)-W_{n}^{*\text{T}}\psi_{n}\left(Z_{n}\right)\|\leq \bar{\varepsilon }$.

**Lemma 3:** The output power of the thruster in this article is large. To make the system input uniformly projected, a more universal analytical method of proportional logarithm projection is designed. The design idea of hyperbolic tangent projection fitting degree saturation filter and the construction method of inequality (Equation 10) in Chen et al. (2022) are used to get inequality (11).


(10) $$\begin{array}{l} -F_{f}\tanh\left(\tau \left(t\right)\right)\leq -\tanh\left(\tau \left(t\right)\right)+1-F_{f} \end{array}$$

(11) $$\begin{array}{l} -\varsigma \frac{1}{\ln\;\left(\theta_{\max}+1\right)}sig\left(\theta \left(t\right)\right)\ln\;\left(|\theta \left(t\right)|+1\right)\\ \leq -\frac{1}{\ln\;\left(\theta_{\max}+1\right)}sig\left(\theta \left(t\right)\right)\ln\;\left(|\theta \left(t\right)|+1\right)+1-\varsigma \end{array}$$

### 4.1. Design failure models

According to the propeller arrangement and motion characteristics of this type of underwater salvage robot, the control torque on its three degrees of freedom is synthesized from the propeller distribution matrix and the four main propellers and is expressed as:


(12) $$\begin{array}{l} \tau =\mathrm{GU} \end{array}$$

**Figure 3:**
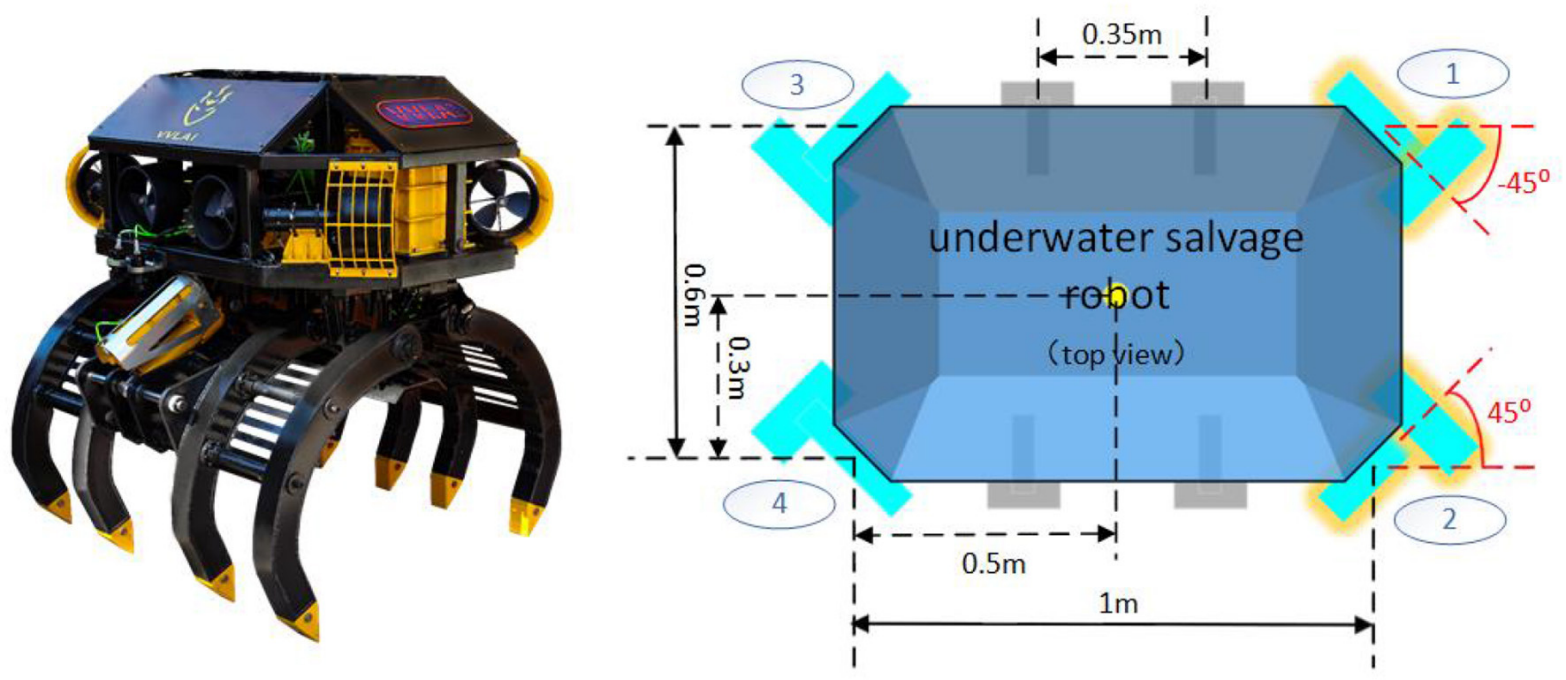
Propeller arrangement.

In the equation $\tau ={\left[\tau_{\text{v}},\tau_{\text{v}},\tau_{\text{r}}\right]}^{\text{T}},\;U={\left[u_{1},u_{2},u_{3},u_{4}\right]}^{\text{T}},\;G=\left[\begin{array}{cccc} 0.5\sqrt{2} & 0.5\sqrt{2} & -0.5\sqrt{2} & -0.5\sqrt{2}\\ 0.5\sqrt{2} & -0.5\sqrt{2} & 0.5\sqrt{2} & -0.5\sqrt{2}\\ 0.4\sqrt{2} & -0.4\sqrt{2} & -0.4\sqrt{2} & 0.4\sqrt{2} \end{array}\right],U$ is the output signals for thrusters 1 to 4, and ***G*** is the configuration matrix for the thrusters determined by the distribution of the thrusters in Figure 3. Since the side thrusters mainly complete the final precise positioning, they do not participate in the power output of the underwater salvage robot. Therefore, the configuration of ***G*** does not consider four small thrusters on the side.

The following fault model is defined according to the failure mode parameters in Table 1.


(13) $$\begin{array}{l} U=\varsigma \theta \left(t\right)+\chi \theta_{\text{s}}\left(t\right) \end{array}$$

In the equation ς = diag[ς_1_, ς_2_, ς_3_, ς_4_] is the failure factor of each propeller, $\theta \left(t\right)={\left[\theta_{1}\left(t\right),\theta_{2}\left(t\right),\theta_{3}\left(t\right),\theta_{4}\left(t\right)\right]}^{\text{T}}$ is the expected thrust of each propeller, χ = diag[χ_1_, χ_2_, χ_3_, χ_4_] is the crankiness generation factor of each propeller, and $\theta_{\text{s}}\left(t\right)={\left[\theta_{\text{s}1}\left(t\right),\theta_{\text{s}2}\left(t\right),\theta_{\text{s}3}\left(t\right),\theta_{\text{s}4}\left(t\right)\right]}^{\text{T}}$ is the unknown paranoid fault of each propeller.

**Table 1:** Propeller failure mode and parameter setting.

**Failure mode**	**ς**	**χ**	**θ_s_(*t*)**	**Cause of failure**
**Normal**	**1**	**0**	**None**	**None**
Invalid	0 < ς < 1	0	None	The propulsion hydraulic system is depressurized and the propulsion paddle is damaged
Stuck	0	1	Exist	Propeller control system damage control failure
Interrupt	0	0	None	Corrosive environment leads to interruption of power supply line and thruster out of operation
Poor contact	0–1 Random parameter	0	None	The contact of control panel is corroded by corrosive gas or leakage liquid

Remark 1: The main faults of the robot are invalid fault or stuck fault, and ς = 1, χ = 0 the thruster is in a healthy state. Some research articles only considered invalid fault (Park and Yoo, 2016) or additive stuck fault (Wang and Han, 2016). This fault model can simulate a variety of fault states at the same time through parameter design and increase the universality of fault-tolerant control algorithm.

Therefore, we improved the dynamic model based on the fault model.


(14) $$\begin{array}{l} MẎ=G\left[\varsigma \theta \left(t\right)+\chi \theta_{\text{s}}\left(t\right)\right]+d\left(t\right)-f\left(x\right) \end{array}$$

In this equation $M=\text{diag}\left[m_{11},m_{22},m_{33}\right],m_{11}=m-X_{\text{i}},\;m_{22}=m-Y_{\text{v}},\;m_{33}=I_{z}-N_{\text{r}},\;d\left(t\right)={\left[\begin{array}{c} \tau_{\text{wv}},\tau_{\text{wv}},\tau_{\text{wr}} \end{array}\right]}^{\text{T}},Y={\left[u,v,r\right]}^{\text{T}},$ and $f\left(x\right)={\left[F_{u}\left(x\right),F_{v}\left(x\right),F_{r}\left(x\right)\right]}^{\text{T}}$

**Figure 4:**
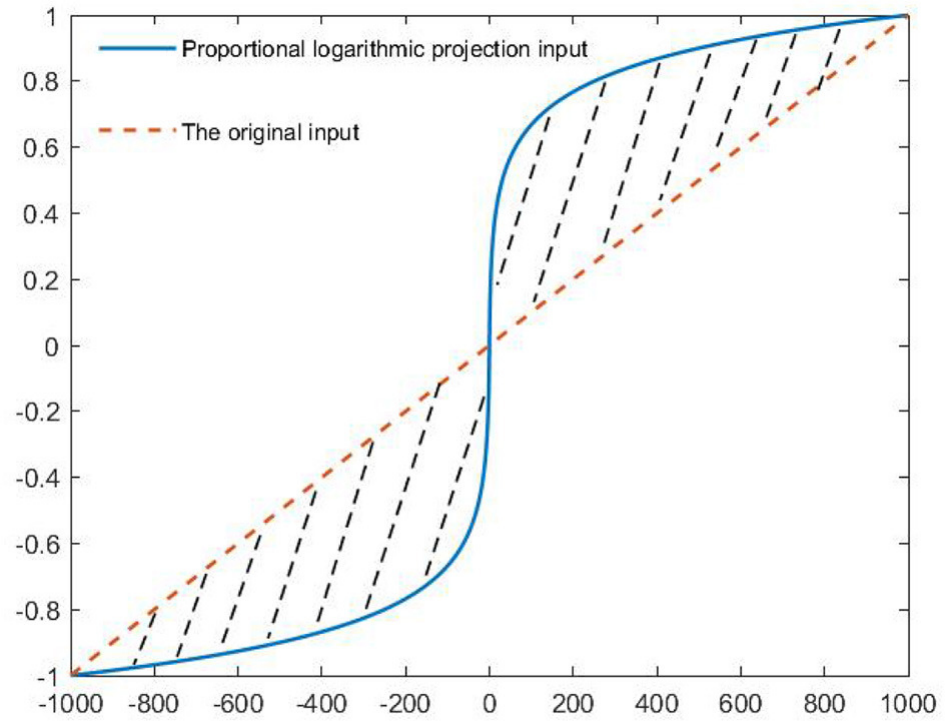
System input projection changes.

To solve the fault factor of system deep coupling in the model, the system input proportional logarithmic projector was designed as shown in Figure 4. The system input was projected in the range of –1 to 1, and then the virtual input was obtained after amplification. The virtual input enhances the compensation of faulty thrusters in the system fault-tolerant control. Substitute the expected input of the system into $\theta_{t}=\theta_{\max}\frac{1}{\ln\;\left(\theta_{\max}+1\right)}sig\left(\theta \left(t\right)\right)\ln\;\left(|\theta \left(t\right)|+1\right)$, we obtain Equation (15).

Remark 2: Compared with the hyperbolic tangent projection method of Chen et al. (2022), the algorithm combines the known saturation attribute characteristics of the thruster into the projection function by using function ln (θ_max_ + 1), which makes the algorithm adaptive to the change in thrust threshold of different thrusters.


(15) $$\begin{array}{l} \dot{Y}=M^{-1}G[\varsigma \theta_{\max}\frac{1}{\ln(\theta_{\max}+1)}sig(\theta (t))\ln(\left|\theta (t)\right|+1)\\ +\chi \theta_{\text{s}}(t)]+M^{-1}d(t)-M^{-1}f(x)\\ \;=M^{-1}G\varsigma \theta_{\max}\frac{1}{\ln(\theta_{\max}+1)}sig(\theta (t))\ln(\left|\theta (t)\right|+1)\\ +M^{-1}G\chi \theta_{\text{s}}(t)+M^{-1}d(t)-M^{-1}f(x) \end{array}$$

(16) $$\begin{array}{l} \varsigma \theta_{\max}\frac{1}{\ln\;\left(\theta_{\max}+1\right)}sig\left(\theta \left(t\right)\right)\ln\;\left(|\theta \left(t\right)|+1\right)\\ \geq \theta_{\max}\left(\frac{1}{\ln\;\left(\theta_{\max}+1\right)}sig\left(\theta \left(t\right)\right)\ln\;\left(|\theta \left(t\right)|+1\right)+\varsigma -1\right) \end{array}$$

In the equation *F*(*x*) = *M*^−1^*f*(*x*), θ_max_ is the absolute value of saturation. According to Lemma 3 and inequality (Equations 16, 17) is obtained to analyze the fault factors of the system model.

Remark 3: The projected input strips the multiplicative fault coefficient of the system into a virtual additive fault through the analytical function (Equation 16). Compared with Hao et al. (2020), it simplifies the coupling relationship between unknown variables and fault coefficients and effectively simplifies the difficulty of control system design and the difficulty of simulation system adjustment.


(17) $$\begin{array}{l} Ẏ\geq M^{-1}G\theta_{\max}\left(\frac{1}{\ln\;\left(\theta_{\max}+1\right)}sig\left(\theta \left(t\right)\right)\ln\;\left(|\theta \left(t\right)|+1\right)+\varsigma -1\right)\\ +M^{-1}G\chi \theta_{\text{s}}\left(t\right)+M^{-1}d\left(t\right)-F\left(x\right)\\ \;\geq \frac{1}{\ln\;\left(\theta_{\max}+1\right)}M^{-1}G\theta_{\max}sig\left(\theta \left(t\right)\right)\ln\;\left(|\theta \left(t\right)|+1\right)+M^{-1}G\theta_{\max}\varsigma \\ \;-M^{-1}G\theta_{\max}+M^{-1}G\chi \theta_{\text{s}}\left(t\right)+M^{-1}d\left(t\right)-F\left(x\right)\\ \;\geq M^{-1}G\theta_{\max}\sigma \theta \left(t\right)+M^{-1}G\theta_{\max}e_{\theta }\\ +M^{-1}G\theta_{\max}\varsigma \\ \;-M^{-1}G\theta_{\max}+M^{-1}G\chi \theta_{\text{s}}\left(t\right)+M^{-1}d\left(t\right)-F\left(x\right) \end{array}$$

In the equation, *e*_θ_ is the projection error, σ is the parser design parameter, $e_{\theta }\text{=}\frac{1}{\ln\;\left(\theta_{\max}+1\right)}sig\left(\theta \left(t\right)\right)\ln\;\left(|\theta \left(t\right)|+1\right)-\sigma \theta_{\text{t}}$. We integrate $M^{-1}G\theta_{\max}e_{\theta }$, $M^{-1}G\theta_{\max}$, $M^{-1}G\chi \theta_{\text{s}}\left(t\right)$, and *M*^−1^*d*(*t*) into the compound uncertainty term *D*(*t*) of the system.


(18) $$\begin{array}{l} D\left(t\right)=M^{-1}G\theta_{\max}e_{\theta }-M^{-1}G\theta_{\max}+M^{-1}G\chi \theta_{\text{s}}\left(t\right)+M^{-1}d\left(t\right) \end{array}$$

### 4.2. Design of system composite disturbance observer


(19) $$\begin{array}{l} Ẏ=\left[\begin{array}{c} \dot{u}\\ \dot{v}\\ ṙ \end{array}\right]=\left[M^{-1}G\theta_{\max}\sigma \theta \left(t\right)+M^{-1}G\theta_{\max}\varsigma +D\left(t\right)\right] \end{array}$$

(20) $$\begin{array}{l} \Delta =Y-Ŷ \end{array}$$

In the equation, Ŷ and Δ are the estimation of the dynamic model *Y* and the estimation error of the dynamic model, respectively.


(21) $$\begin{array}{l} \dot{Ŷ}=\left[\begin{array}{c} \dot{u}\\ \dot{v}\\ ṙ \end{array}\right]=\left[M^{-1}G\theta_{\max}\sigma \theta \left(t\right)+M^{-1}G\theta_{\max}\varsigma -F\left(x\right)+\hat{D}\left(t\right)\right] \end{array}$$

Set the sliding mode switching surface to *S*.


(22) $$\begin{array}{l} S=\Delta +E{\dot{\Delta }}^{\eta } \end{array}$$

In this equation, $S={\left[S_{\text{u}},S_{\text{v}},S_{\text{r}}\right]}^{\text{T}},\Delta ={\left[\Delta_{\text{u}},\Delta_{\text{v}},\Delta_{\text{r}}\right]}^{\text{T}},E=\mathrm{diag}\left\{b_{1},b_{2},b_{3}\right\},\;\eta =\frac{p}{q},p,q$ are positive odd numbers and satisfy 1 < η < 2.


(23) $$\begin{array}{l} \dot{\hat{D}}\left(t\right)=\frac{1}{\eta }E^{-1}{\dot{\Delta }}^{2-\eta }+\varepsilon S+\left(\gamma +Ĥ\right)sign\left(S\right) \end{array}$$

(24) $$\begin{array}{l} {\dot{Ĥ}}_{\text{v}}=\eta \zeta \overset{3}{\underset{i}{\sum }}E_{i}F_{i}|S_{i}| \end{array}$$

In the equation, *H* is an upper bound on Ḋ(*t*), $\hat{D}\left(t\right)$ and Ĥ are estimates of *D*(*t*) and *H*, respectively, *E* = diag{*b*_1_, *b*_2_, *b*_3_}. *b*_1_, *b*_2_, and *b*_3_ greater than zero, $F=\mathrm{diag}\left[{\dot{\Delta }}_{\text{u}}^{\eta -1},{\dot{\Delta }}_{\text{v}}^{\eta -1},{\dot{\Delta }}_{\text{r}}^{\eta -1}\right]$. ε, γ, and ζ are normal numbers. By using Lemma 1, taking the derivative of Equation (20), we can get:


(25) $$\begin{array}{l} \dot{\Delta }=Ẏ-\dot{Ŷ} \end{array}$$

Substituting (Equations 19, 21) into Equation (25), we can obtain:


(26) $$\begin{array}{l} \dot{\Delta }=Ẏ-\dot{Ŷ}=D\left(t\right)-\hat{D}\left(t\right) \end{array}$$

(27) $$\begin{array}{l} \ddot{\Delta }=Ḋ\left(t\right)-\dot{\hat{D}}\left(t\right) \end{array}$$

Taking the derivative of *S* leads to:


(28) $$\begin{array}{l} \begin{array}{c} Ṡ=\dot{\Delta }+\eta EF\ddot{\Delta }\\ \;=\dot{\Delta }+\eta EF(Ḋ\left(t\right)-\dot{\hat{D}}\left(t\right) \end{array} \end{array}$$

Substituting (Equation 23) into Equation (28), we can get:


(29) $$\begin{array}{l} Ṡ=\eta EF\left[Ḋ\left(t\right)-\varepsilon S-\left(\gamma +Ĥ\right)sign\left(S\right)\right] \end{array}$$

The stability of the observer is analyzed, and the Lyapunov function is set as,


(30) $$\begin{array}{l} V_{\text{d}}=\frac{1}{2}S^{\text{T}}S+\frac{1}{2\zeta }{\tilde{H}}^{2} \end{array}$$

Taking the derivative of *V*_μ_v__ leads to:


(31) $$\begin{array}{l} {\dot{V}}_{\text{d}}=S^{\text{T}}\dot{S}-\frac{1}{\zeta }\tilde{H}\dot{\hat{H}}\\ \;=-\varepsilon \eta S^{\text{T}}EFS-\gamma \eta \sum_{i=1}^{3}E_{i}F_{i}\left|S_{i}\right|-\frac{1}{\zeta }\tilde{H}\dot{\hat{H}}+\eta \sum_{i=1}^{3}E_{i}F_{i}(S_{i}\dot{D}(t)\\ -\left|S_{i}\right|\hat{H})\\ \;\leq -\varepsilon \eta S^{\text{T}}EFS-\gamma \eta \sum_{i=1}^{3}E_{i}F_{i}\left|S_{i}\right|-\frac{1}{\zeta }\tilde{H}\dot{\hat{H}}+\eta \sum_{i=1}^{3}E_{i}F_{i}(\left|S_{i}\right|\dot{D}(t)\\ -\left|S_{i}\right|\hat{H})\\ \;=-\varepsilon \eta S^{\text{T}}EFS-\gamma \eta \sum_{i=1}^{3}E_{i}F_{i}\left|S_{i}\right|-\frac{1}{\zeta }\tilde{H}\dot{\hat{H}}+\eta \sum_{i=1}^{3}E_{i}F_{i}\left|S_{i}\right|\tilde{H} \end{array}$$

Substituting (Equation 24) into Equation (31), we can get:


(32) $$\begin{array}{l} {\dot{V}}_{\text{d}}\leq -\varepsilon \eta S^{\text{T}}EFS-\gamma \eta \overset{3}{\underset{i=1}{\sum }}E_{i}F_{i}|S_{i}| \end{array}$$

The observer is stable because of ${\dot{V}}_{d}<0$, and the system state converges to the sliding mode surface in finite time *S* = 0 (Feng et al., 2002), so the system can fully estimate *D*(*t*) in finite time, $D\left(t\right)=\hat{D}\left(t\right)$.

### 4.3. Design of projection analytical overdrive controller for underwater fishing robot

**Assumption 1:** The weight W^*^ of the Radial Basis Function (RBF) neural network used to approximate the unknown vector is bounded. There are positive constants *W*_*M*_, and $\|W^{*}\|\leq W_{M}$, ε is the neural network approximation error.

**Assumption 2:** The external environmental interference, system velocity, and acceleration are bounded and smooth in the range of *t* ∈ *R*^+^, Interference force / torque τ_wu_, τ_wv_, τ_wr_ acting on the robot itself. Suppose there are several unknown constants, τ_*wu*_max__ > 0, τ_*wv*_max__ > 0, τ_*wr*_max__ > 0, consistent with |τ_*wu*_| < τ_*wu*_max__, |τ_*wv*_| < τ_*wv*_max__, and |τ_*wr*_| < τ_*wr*_max__.

System velocity error (Equation 33),


(33) $$\begin{array}{l} \begin{array}{c} {Ẏ}_{\text{e}}={Ẏ}_{\text{d}}-Ẏ={Ẏ}_{\text{d}}-M^{-1}G\varsigma \theta_{\max}\frac{1}{\ln\;\left(\theta_{\max}+1\right)}sig\left(\theta \left(t\right)\right)\ln\;\left(|\theta \left(t\right)|+1\right)\\ \;-M^{-1}G\chi \theta_{\text{s}}\left(t\right)-M^{-1}d\left(t\right)+F\left(x\right) \end{array} \end{array}$$

In the equation, $Y_{\text{e}}={\left[u_{\text{e}},v_{\text{e}},r_{\text{e}}\right]}^{\text{T}},u_{\text{e}}=u_{\text{d}}-u,v_{\text{e}}=v_{\text{d}}-v,r_{\text{e}}=r_{\text{d}}-r$. The RBF neural network algorithm is designed to approximate the system dynamic uncertainty,


(34) $$\begin{array}{l} F\left(x\right)=W^{*\text{T}}\psi \left(z\right)+\varepsilon \end{array}$$

(35) $$\begin{array}{l} \begin{array}{c} {Ẏ}_{\text{e}}={Ẏ}_{\text{d}}-Ẏ={Ẏ}_{\text{d}}-M^{-1}G\varsigma \theta_{\max}\frac{1}{\ln\;\left(\theta_{\max}+1\right)}sig\left(\theta \left(t\right)\right)\ln\;\left(|\theta \left(t\right)|+1\right)\\ \;-M^{-1}G\chi \theta_{\text{s}}\left(t\right)-M^{-1}d\left(t\right)+W^{*\text{T}}\psi \left(z\right)+\varepsilon \end{array} \end{array}$$

Lyapunov function is designed for system velocity error


(36) $$\begin{array}{l} V_{\text{v}}=\frac{1}{2}{Y_{\text{e}}}^{\text{T}}Y_{\text{e}}+\overset{m}{\underset{i=1}{\sum }}\frac{{\tilde{\varsigma }}_{i}^{2}\left(t\right)}{2y_{i}}+\overset{n}{\underset{i=1}{\sum }}\frac{{\tilde{\Theta }}_{i}^{2}\left(t\right)}{2} \end{array}$$

In the equation, *m* = 4, *n* = 3, taking the derivative of Lyapunov (Equation 36), we obtain:


(37) $$\begin{array}{l} {\dot{V}}_{\text{v}}={Y_{\text{e}}}^{\text{T}}{Ẏ}_{\text{e}}+\overset{m}{\underset{i=1}{\sum }}\frac{{\tilde{\varsigma }}_{i}\left(t\right){\dot{\hat{\varsigma }}}_{i}\left(t\right)}{y_{i}}+\overset{n}{\underset{i=1}{\sum }}{\tilde{\Theta }}_{i}\left(t\right){\dot{\hat{\Theta }}}_{i}\left(t\right) \end{array}$$

(38) $$\begin{array}{l} {\dot{V}}_{\text{v}}=Y_{\text{e}}{\;}^{\text{T}}[{\dot{Y}}_{\text{d}}-M^{-1}G\varsigma \theta_{\max}\frac{1}{\ln(\theta_{\max}+1)}sig(\theta (t))\ln(\left|\theta (t)\right|+1)\\ \;-M^{-1}G\chi \theta_{\text{s}}(t)-M^{-1}d(t)+W^{*\text{T}}\psi (z)+\varepsilon ]\\ \;+\sum_{i=1}^{m}\frac{{\tilde{\varsigma }}_{i}(t){\dot{\hat{\varsigma }}}_{i}(t)}{y_{i}}+\sum_{i=1}^{n}{\tilde{\Theta }}_{i}(t){\dot{\hat{\Theta }}}_{i}(t) \end{array}$$

Substituting (Equation 17) into Equation (38), the following can be obtained:


(39) $$\begin{array}{l} {\dot{V}}_{\text{v}}\leq Y_{\text{e}}{\;}^{\text{T}}[{\dot{Y}}_{\text{d}}-M^{-1}G\theta_{\max}\sigma \theta (t)-M^{-1}G\theta_{\max}e_{\theta }-M^{-1}G\theta_{\max}\varsigma \\ \;+M^{-1}G\theta_{\max}-M^{-1}G\chi \theta_{\text{s}}(t)-M^{-1}d(t)+W^{*\text{T}}\psi (z)+\varepsilon ]\\ \;+\sum_{i=1}^{m}\frac{{\tilde{\varsigma }}_{i}(t){\dot{\hat{\varsigma }}}_{i}(t)}{y_{i}}+\sum_{i=1}^{n}{\tilde{\Theta }}_{i}(t){\dot{\hat{\Theta }}}_{i}(t)\\ \;\leq Y_{\text{e}}{\;}^{\text{T}}[{\dot{Y}}_{\text{d}}-M^{-1}G\theta_{\max}\sigma \theta (t)-M^{-1}G\theta_{\max}\varsigma -D(t)\\ +W^{*\text{T}}\psi (z)+\varepsilon ]\\ \;+\sum_{i=1}^{m}\frac{{\tilde{\varsigma }}_{i}(t){\dot{\hat{\varsigma }}}_{i}(t)}{y_{i}}+\sum_{i=1}^{n}{\tilde{\Theta }}_{i}(t){\dot{\hat{\Theta }}}_{i}(t) \end{array}$$

According to assumption 1 and assumption 2, combined with the design idea of the robust adaptive method for depth information in the literature, RBF neural network approximation technology and minimum learning parameter (MLP) technology are adopted to obtain:


(40) $$\begin{array}{l} \|{W_{n}}^{*\text{T}}\psi_{n}\left(z\right)+\varepsilon_{n}\|\leq \|{W_{n}}^{*\text{T}}\|\|\psi_{{\;}_{n}}\left(z\right)\|+|\varepsilon_{n}|=\Theta \phi \left(z\right) \end{array}$$

In the equation, $\Theta =\max\left\{\left\|W_{n}^{*\text{T}}\|,|\varepsilon_{n}\right|\right\};\;\phi \left(z\right)=\|\psi_{n}\left(z\right)\|+1$. According to Equation (40), the adaptive learning parameters are significantly reduced.


(41) $$\begin{array}{l} \begin{array}{c} {\dot{V}}_{\text{v}}\leq {Y_{\text{e}}}^{\text{T}}\left\{{Ẏ}_{\text{d}}-\left[M^{-1}G\theta_{\max}\sigma \theta \left(t\right)+M^{-1}G\theta_{\max}\varsigma +D\left(t\right)-\Theta \phi \left(z\right)\right]\right\}\\ \;+\overset{m}{\underset{i=1}{\sum }}\frac{{\tilde{\varsigma }}_{i}\left(t\right){\dot{\hat{\varsigma }}}_{i}\left(t\right)}{y_{i}}+\overset{n}{\underset{i=1}{\sum }}{\tilde{\Theta }}_{i}\left(t\right){\dot{\hat{\Theta }}}_{i}\left(t\right) \end{array} \end{array}$$

(42) $$\begin{array}{l} {Y_{\text{e}}}^{\text{T}}\Theta \phi \left(z\right)\leq \overset{n}{\underset{i=1}{\sum }}\left[a_{i}\Theta_{i}{\phi_{i}}^{2}\left(z\right){\left|{Y_{\text{ei}}}^{\text{T}}\right|}^{2}+\frac{\Theta_{i}}{4a_{i}}\right] \end{array}$$

(43) $$\begin{array}{l} \begin{array}{c} {\dot{V}}_{\text{v}}\leq {Y_{\text{e}}}^{\text{T}}\left\{{Ẏ}_{\text{d}}-\left[M^{-1}G\theta_{\max}\sigma \theta \left(t\right)+M^{-1}G\theta_{\max}\varsigma +D\left(t\right)\right]\right\}\\ \;+\overset{n}{\underset{i=1}{\sum }}\left[a_{i}\Theta_{i}{\phi_{i}}^{2}\left(z\right){\left|{Y_{\text{ei}}}^{\text{T}}\right|}^{2}+\frac{\Theta_{i}}{4a_{i}}\right]+\overset{m}{\underset{i=1}{\sum }}\frac{{\tilde{\varsigma }}_{i}\left(t\right){\dot{\hat{\varsigma }}}_{i}\left(t\right)}{y_{i}}+\overset{n}{\underset{i=1}{\sum }}{\tilde{\Theta }}_{i}\left(t\right){\dot{\hat{\Theta }}}_{i}\left(t\right) \end{array} \end{array}$$

Design fault-tolerant control (Equation 44) and fault-tolerant adaptive (Equations 45, 46),


(44) $$\begin{array}{l} \theta \left(t\right)=\frac{G^{+}M}{\sigma \theta_{\max}}\left[\delta Y_{\text{e}}+u_{\text{p}}-\hat{D}\left(t\right)+{Ẏ}_{\text{d}}-M^{-1}G\theta_{\max}\hat{\varsigma }\right]\\ +\frac{G^{+}M}{\sigma \theta_{\max}}\overset{n}{\underset{i=1}{\sum }}\left[a_{i}{\hat{\Theta }}_{i}{\phi_{i}}^{2}\left(z\right)|{Y_{\text{ei}}}^{\text{T}}|\right] \end{array}$$

(45) $$\begin{array}{l} {\dot{\hat{\varsigma }}}_{i}=-y_{i}\|{Y_{\text{e}}}^{\text{T}}\|\|M^{-1}\|\|G_{i}\|\theta_{\max}+\psi {\hat{\varsigma }}_{i} \end{array}$$

(46) $$\begin{array}{l} {\dot{\hat{\Theta }}}_{i}=a_{i}{\phi_{i}}^{2}\left(z\right)|{Y_{\text{ei}}}^{\text{T}}{|}^{2}+\chi_{i}{\hat{\Theta }}_{i} \end{array}$$

In the equation, $u_{\text{p}}={\left[e_{1}\cos\psi ,e_{2}\cos\psi ,e_{3}\right]}^{\text{T}}$. δ, ψ, γ, *a*, and χ are the normal variables to be designed for gain in controller design.

Design controller event trigger conditions are as follows:


(47) $$\begin{array}{l} \theta \left(t\right)=L\left(t_{k}\right),\;\forall t\in \left[t_{k},t_{k+1}\right) \end{array}$$

(48) $$\begin{array}{l} t_{k+1}=\inf\left\{t>t_{k}\|e\left(t\right)|\geq |u\left(t\right)|\right\},t_{1}=0 \end{array}$$

In the equation, *e*(*t*) = *L*(*t*)−θ(*t*) represents the input error of the control rate after the event trigger mechanism. $t_{k},k\in z^{+}$ represents the time when the trigger condition is triggered, and the control signal θ(*t*) at that moment is applied to the system. The control signal keeps *L*(*t*) unchanged at *t* ∈ [*t*_*k*_, *t*_*k*+1_) time.

Substitute (Equation 44) into Equation (43) to simplify, we get:


(49) $$\begin{array}{l} \begin{array}{c} {\dot{V}}_{\text{v}}\leq {Y_{\text{e}}}^{\text{T}}\left\{-\delta Y_{\text{e}}-u_{\text{p}}-\tilde{D}\left(t\right)\right\}+\|Y_{\text{e}}\|\|M^{-1}\|\overset{m}{\underset{i=1}{\sum }}\|G_{i}\|\theta_{i\max}\|{\tilde{\varsigma }}_{i}\|\\ \;-\overset{n}{\underset{i=1}{\sum }}\left[a_{i}{\tilde{\Theta }}_{i}{\phi_{i}}^{2}\left(z\right){\left|{Y_{\text{ei}}}^{\text{T}}\right|}^{2}+\frac{\Theta_{i}}{4a_{i}}\right]+\overset{m}{\underset{i=1}{\sum }}\frac{{\tilde{\varsigma }}_{i}\left(t\right){\dot{\hat{\varsigma }}}_{i}\left(t\right)}{y_{i}}+\overset{n}{\underset{i=1}{\sum }}{\tilde{\Theta }}_{i}\left(t\right){\dot{\hat{\Theta }}}_{i}\left(t\right) \end{array} \end{array}$$

(50) $$\begin{array}{l} {\tilde{\varsigma }}_{i}\left(t\right){\hat{\varsigma }}_{i}\left(t\right)={\tilde{\varsigma }}_{i}\left(t\right)\left(\varsigma_{i}\left(t\right)-{\tilde{\varsigma }}_{i}\left(t\right)\right)\leq -\frac{{{\tilde{\varsigma }}_{i}}^{2}\left(t\right)}{2}+\frac{{\varsigma_{i}}^{2}\left(t\right)}{2} \end{array}$$

(51) $$\begin{array}{l} {\tilde{\Theta }}_{i}\left(t\right){\hat{\Theta }}_{i}\left(t\right)={\tilde{\Theta }}_{i}\left(t\right)\left(\Theta_{i}\left(t\right)-{\hat{\Theta }}_{i}\left(t\right)\right)\leq -\frac{{\tilde{\Theta }}_{i}{\left(t\right)}^{2}}{2}+\frac{\Theta_{i}{\left(t\right)}^{2}}{2} \end{array}$$

Because the terminal sliding mode observer makes $D\left(t\right)=\hat{D}\left(t\right)$ in finite time, $\tilde{D}\left(t\right)=0$. By substituting the adaptive (Equations 45, 46), inequality (Equation 49) simplifies to:


(52) $$\begin{array}{l} {\dot{V}}_{\text{v}}\leq {Y_{\text{e}}}^{\text{T}}\left\{-\delta Y_{\text{e}}-u_{\text{p}}\right\}-\overset{m}{\underset{i=1}{\sum }}\frac{\psi_{i}{{\tilde{\varsigma }}_{i}}^{2}\left(t\right)}{2y_{i}}+\overset{m}{\underset{i=1}{\sum }}\frac{\psi_{i}{\varsigma_{i}}^{2}\left(t\right)}{2y_{i}}\\ -\overset{n}{\underset{i=1}{\sum }}\frac{\chi_{i}{\tilde{\Theta }}_{i}{\left(t\right)}^{2}}{2}+\overset{n}{\underset{i=1}{\sum }}\frac{\chi_{i}\Theta_{i}{\left(t\right)}^{2}}{2} \end{array}$$

(53) $$\begin{array}{l} P=V+V_{\text{v}} \end{array}$$

Taking the derivative of Equation (53), we can get:


(54) $$\begin{array}{l} \begin{array}{c} Ṗ=\dot{V}+{\dot{V}}_{\text{v}}\leq {Y_{\text{e}}}^{\text{T}}u_{\text{p}}-k_{1}{e_{1}}^{2}-k_{3}{e_{2}}^{2}-k_{5}{e_{3}}^{2}+{Y_{\text{e}}}^{\text{T}}\left\{-\delta Y_{\text{e}}-u_{\text{p}}\right\}\\ \;-\overset{m}{\underset{i=1}{\sum }}\frac{\psi_{i}{{\tilde{\varsigma }}_{i}}^{2}\left(t\right)}{2y_{i}}+\overset{m}{\underset{i=1}{\sum }}\frac{\psi_{i}{\varsigma_{i}}^{2}\left(t\right)}{2y_{i}}-\overset{n}{\underset{i=1}{\sum }}\frac{\chi_{i}{\tilde{\Theta }}_{i}{\left(t\right)}^{2}}{2}+\overset{n}{\underset{i=1}{\sum }}\frac{\chi_{i}\Theta_{i}{\left(t\right)}^{2}}{2}\\ \;\leq -k_{1}{e_{1}}^{2}-k_{3}{e_{2}}^{2}-k_{5}{e_{3}}^{2}-\delta {Y_{\text{e}}}^{\text{T}}Y_{\text{e}}\\ \;-\overset{m}{\underset{i=1}{\sum }}\frac{\psi_{i}{{\tilde{\varsigma }}_{i}}^{2}\left(t\right)}{2y_{i}}+\overset{m}{\underset{i=1}{\sum }}\frac{\psi_{i}{\varsigma_{i}}^{2}\left(t\right)}{2y_{i}}-\overset{n}{\underset{i=1}{\sum }}\frac{\chi_{i}{\tilde{\Theta }}_{i}{\left(t\right)}^{2}}{2}+\overset{n}{\underset{i=1}{\sum }}\frac{\chi_{i}\Theta_{i}{\left(t\right)}^{2}}{2}\\ \;\leq -\mu P+C \end{array} \end{array}$$

In the equation, μ = {*k*_1_, *k*_3_, *k*_5_, δ, 0.5ψ_*i*_/*y*_*i*_, 0.5χ_*i*_}, $C=\overset{m}{\underset{i=1}{\sum }}\frac{\psi_{i}{\varsigma_{i}}^{2}\left(t\right)}{2y_{i}}+\overset{n}{\underset{i=1}{\sum }}\frac{\chi_{i}\Theta_{i}{\left(t\right)}^{2}}{2}$.

No Zeno phenomenon is proved, when t^*^ > 0, ∀*k* ∈ *z*^+^, $t_{k+1}-t_{k}\geq t^{*}$.


(55) $$\begin{array}{l} e\left(t\right)=L\left(t\right)-\theta \left(t\right),\forall t\in \left[t_{k},t_{k+1}\right) \end{array}$$

(56) $$\begin{array}{l} \frac{d}{dt}|e|=\frac{d}{dt}{\left(e*e\right)}^{\frac{1}{2}}=sign\left(e\right)ė\leq |\dot{L}| \end{array}$$

According to the control law, $\dot{L}$ is a continuously differentiable function composed of *Y*, $\hat{\varsigma }$, and $\hat{\Theta }$. Therefore, there must exist a constant Ξ>0, satisfying the condition $|\dot{L}|\leq \Xi$. If *e*(*t*_*k*_), $\underset{t\to t_{k+1}}{\lim}$ *e*(*t*) = Υ, there must be some positive constant t^*^ that satisfies condition $t^{*}\geq \frac{\Upsilon }{\Xi }$. The Zeno phenomenon does not occur.

Remark 4: Consider the component wear caused by the frequent change in power output of the thruster in the actual project, and the heavy underwater salvage robot still needs to ensure the effectiveness of the thruster in the state of unknown fault to prevent the occurrence of runaway phenomenon. Drawing lessons from the control design of article (Zhu et al., 2021), the power output is processed by event trigger to protect the thruster.

**Theorem 1:** Under assumption 2, aiming at the trajectory tracking control problem of underwater salvage robot with model dynamic uncertainty, unknown time-varying coincidence disturbance, and thruster fault, the control law (Equations 5–7, 47) and its adaptive law (Equations 45, 46) are designed to enable the underwater salvage robot to track the desired position and pose and to ensure that other signals of the closed-loop trajectory tracking control system are bounded. By selecting the appropriate controller design parameters, the tracking error of the underwater salvage robot can be adjusted to a smaller neighborhood.

**Proof**. Solving formula (Equation 54), we get:


(57) $$\begin{array}{l} 0\leq P\leq \frac{C}{\mu }+\left[P\left(0\right)-\frac{C}{\mu }\right]e^{-\mu t} \end{array}$$

In the equation, *P*(0) is the initial value of *P*. According to formula (Equation 57), when $\underset{t\to \infty }{\lim}P\leq \frac{C}{\mu }$, it proves that *P* is bounded. The boundedness of *P* can also prove that $e_{1},e_{2},e_{3},u_{\text{e}},v_{\text{e}},r_{\text{e}},{\tilde{\varsigma }}_{i},{\tilde{\Theta }}_{i}$ are bounded. Therefore, *u*_*d*_, *v*_*d*_, and *r*_*d*_ are also bounded. Combined with assumption 1, assumption 2, and Lemma 2, the kernel function is bounded by the approximation principle of the RBF neural network, so ϕ(*z*) is also bounded, and the designed control law (Equation 44), the adaptive law (Equations 45, 46) is bounded. Therefore, all signals in the closed-loop trajectory tracking fault-tolerant control system are bounded.

## 5. System simulation analysis

To prove the effectiveness and stability of the designed controller, the following ladder simulation system is designed. The basic parameters of the system adopt the model parameters of the Shandong future robot gravity salvaging robot (mine-1): m_11_ = 2345.31, m_22_ = 2900.8, m_33_ = 1373.916, θ_max_ = 1, 000. The PEFC control scheme in this paper is compared with the traditional adaptive overdrive fault tolerant controller (AOFC) (Hao et al., 2020) for underwater salvage robot.

After the underwater salvage robot is lowered by the carrier ship after determining the general orientation, it relies on the underwater salvage robot's own camera for search and positioning, and the search trajectory is a trapezoidal trajectory to enhance the search efficiency, and the trapezoidal reference trajectory equation is set in this study as follows:


(58) $$\begin{array}{l} y_{\text{d}}\left(t\right)=\{\begin{array}{cc} 10 & t\leq 47\\ \sqrt{100-{\left(t-47\right)}^{2}} & 47<t\leq 53\\ \frac{65-t}{1.5} & 53<t\leq 62\\ 10-\sqrt{100-{\left(t-68\right)}^{2}} & 62<t\leq 68\\ 0 & 68<t\leq 112\\ 10-\sqrt{100-{\left(t-112\right)}^{2}} & 112<t\leq 118\\ \frac{t-115}{1.5} & 118<t\leq 127\\ \sqrt{100-{\left(t-133\right)}^{2}} & 127<t\leq 133\\ 10 & 133<t\leq 200 \end{array} \end{array}$$

(59) $$\begin{array}{l} x_{\text{d}}\left(t\right)=t \end{array}$$

(60) $$\begin{array}{l} \theta_{\text{d}}\left(t\right)=\arctan\left(\left(dy/dt\right)\;\left(dx/dt\right)\right) \end{array}$$

Compound disturbance of ocean current $D\left(t\right)={\left[\tau_{\text{wu}},\tau_{\text{wv}},\tau_{\text{wr}}\right]}^{\text{T}}={\left[30\sin\left(2t\right)+p_{1},30\cos\left(2t\right)+p_{2},\text{30sin(t)}+p_{3}\right]}^{\text{T}}$. In the equation *p*_1_, *p*_2_, and *p*_3_ are white noise perturbations with an intensity of 100 and an interval of 1 (Zhang, 2022).

Observer parameters:

**Table:** 

*b*_1_	*b*_2_	*b*_3_	ε_1_	ε_2_	ε_3_	γ_1_	γ_2_	γ_3_	η	ζ
1.13	1.52	1.52	100	112	120	0.2	0.8	0.8	1.36	0.1

Controller design parameters:

**Table:** 

*k*_1_	40	σ_1_	0.1	*a*_3_	0.05	*y*_3_	20	ψ_2_	0.01
*k*_3_	2	σ_2_	0.1	χ_1_	0.02	*y*_4_	100	ψ_3_	0.01
*k*_5_	10	σ_3_	0.1	χ_2_	0.02	χ_1_	0.02	ψ_4_	0.01
δ_1_	12,000	σ_4_	0.1	χ_3_	0.02	χ_2_	0.02		
δ_2_	10,000	*a*_1_	0.005	*y*_1_	10	χ_3_	0.02		
δ_3_	16,000	*a*_2_	0.0005	*y*_2_	150	ψ_1_	0.01		

*k* and δ parameters control system overshoot and stabilization speed, σ parameter regulates the mobilization threshold of the thruster in case of failure, ψ, γ, *a* and χ adjustment system parameters compensates for dynamic uncertainties and external ocean current composite interference.

**Figure 5:**
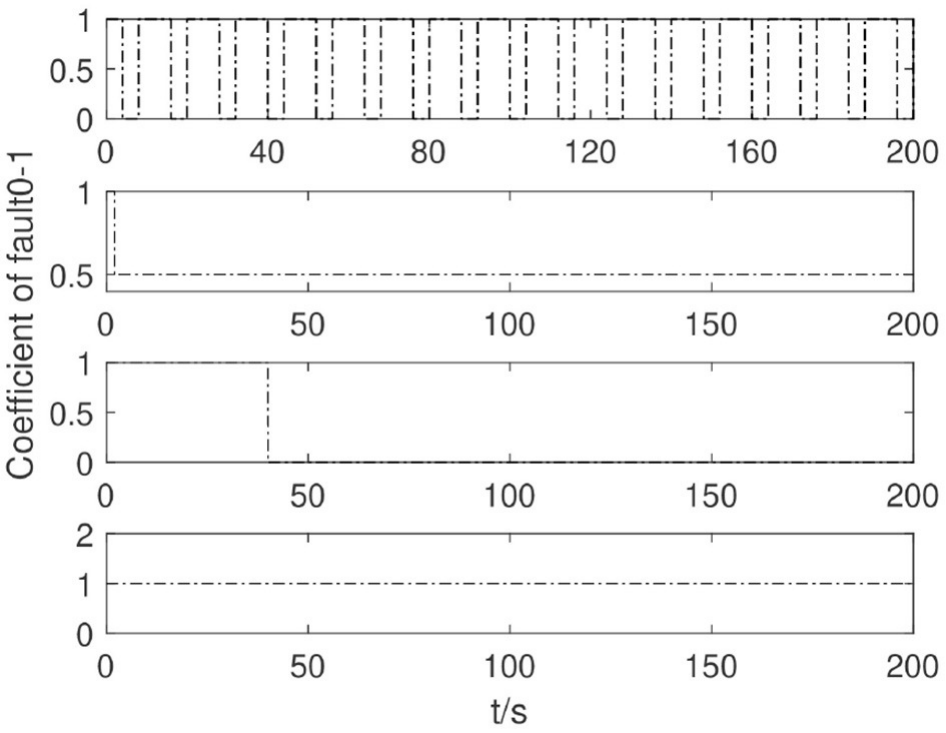
Propeller failure curve.

According to the form of the fault considered in the study, the maximization of the fault is designed to demonstrate the effectiveness of the proposed algorithm. The simulation considers the fault situation as shown in Figure 5. When the equipment work begins, the No. 1 thruster has a poor contact fault, No. 2 thruster failed 50% at time *t* ≥ 2*s*, and No. 3 thruster at moment *t* ≥ 40*s* has a stuck fault paranoid signal of 50*N*. The, we control the whole process. There was no failure on No. 4 thruster.

**Figure 6:**
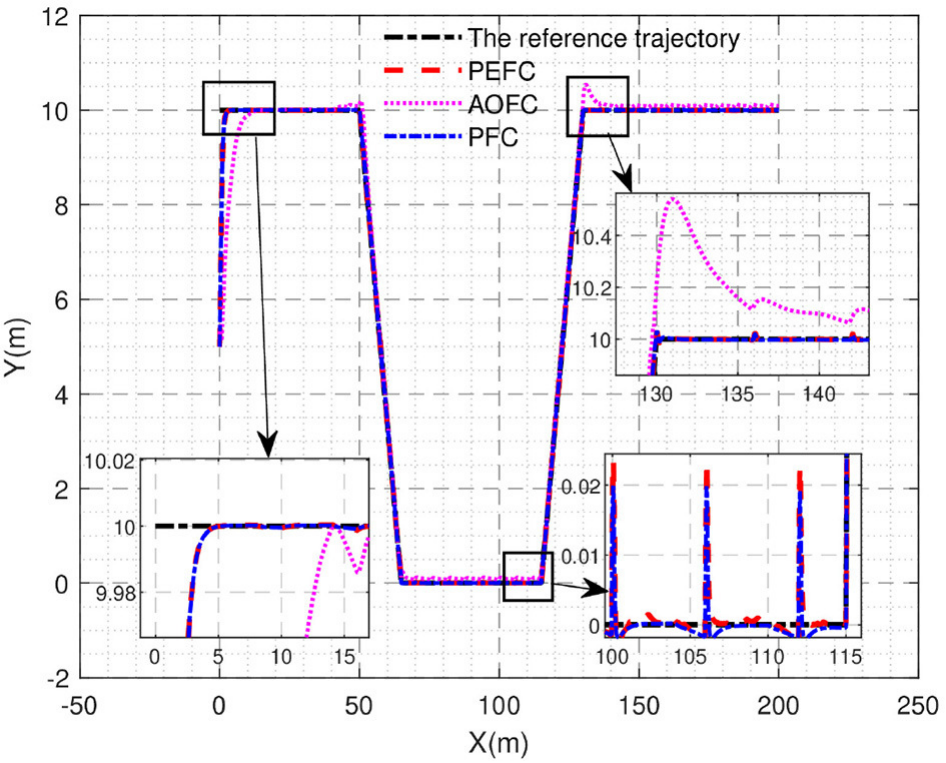
Trajectory tracking curve.

The underwater salvage robot can follow the desired trajectory in the case of three thruster failures as shown in Figure 6, but the PEFC controller and PFC controller can reach the desired trajectory smoothly and track accurately within the bounded range of the desired trajectory even if the propulsion system is not used at full power, while the AOFC controller is relatively slow. In the first turn of the trapezoidal trajectory, when all four thruster design faults have appeared, it can be seen that the PEFC controller and the PFC controller control effect can more clearly and quickly control the motion trajectory of the underwater salvage robot in the desired motion trajectory near; however, in the AOFC controller, because the fault signal in the system signal depth coupling can not be stripped, there is poor control sensitivity of the adaptive controller, the trajectory. The deviation from the desired trajectory is reflected on the PEFC and PFC controllers, and the control accuracy is low compared with the algorithm in this paper. The effect of adding event-triggered control to the algorithm is not significantly different, only in the process of steady-state tracking after event-triggered processing of the PEFC controller has a relatively large jitter compared to the PFC controller.

**Figure 7:**
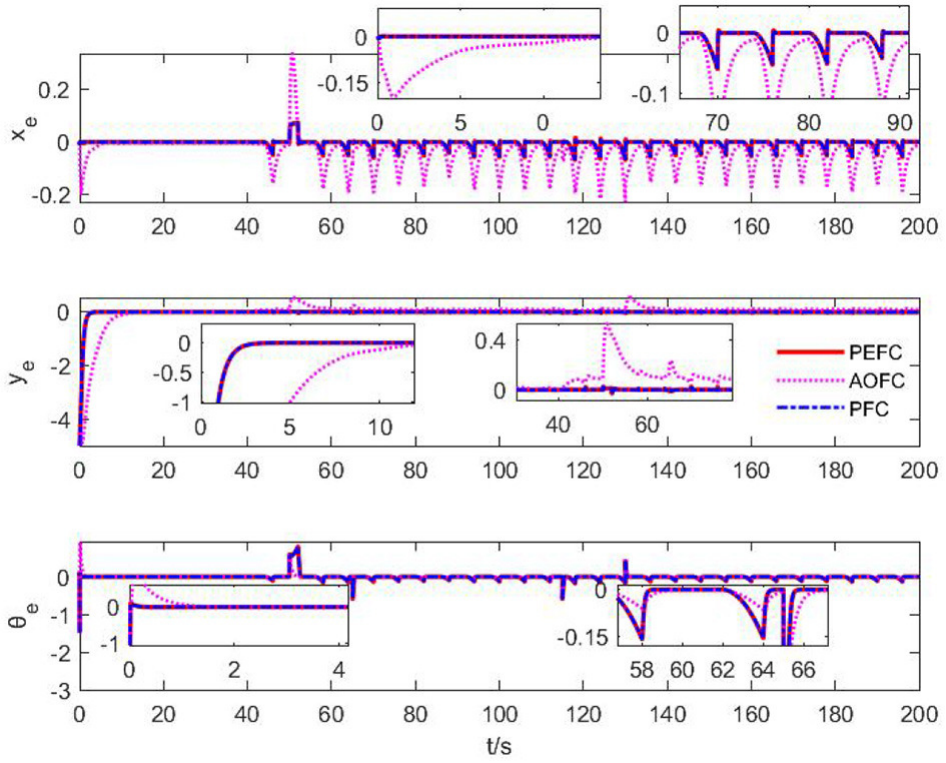
Position and attitude error curve.

The position and attitude error of the underwater vehicle is shown in Figure 7. In the initial adjustment stage of the *x*_*e*_ curve, the PEFC controller is stably controlled in the bounded interval near 0, while the AOFC controller will overshoot and stabilize in the bounded interval near 0 in approximately 5 s, and in the middle tracking stage. Both the PEFC controller and the AOFC controller can stably and accurately track the desired position information in the case of poor contact failure of the No. 1 thruster and the 50% failure of the No. 2 thruster, but after the paranoid interference occurs in the system after 40 s, both the PEFC controller and the AOFC controller fluctuate, but the PEFC controller can still keep stable tracking in the steady tracking state and the fluctuation is much smaller than that of the AOFC controller. In the initial adjustment stage of the *y*_*e*_ curve, the PEFC controller is stable in the bounded neighborhood of 0 in 5 s; then, the AOFC controller is stable in the bounded neighborhood of 0 in 12 s. In the mid-term tracking phase, both the PEFC controller and the AOFC controller can stably and accurately track the expected position information in the case of poor contact failure of the No. 1 thruster and the 50% failure of the No. 2 thruster, but the PEFC controller can still track stably and maintain a small jitter after 40 s of paranoid interference in the system, but the AOFC controller has steady-state error and jitter. In the initial adjustment stage of the θ_*e*_ curve, the control of the PEFC controller can correct the attitude angle error at the start stage, while the AOFC corrects the attitude angle error after 2 s, and the control effect in the middle tracking stage is clearly the same. From the earlier analysis, we can see that the PEFC control strategy designed in this article is better than the comparative AOFC control scheme in control accuracy and speed. The PEFC control scheme which uses projection analysis to separate the fault coupling of the system has a better control effect when the system jam fault is affected by bias interference.

**Figure 8:**
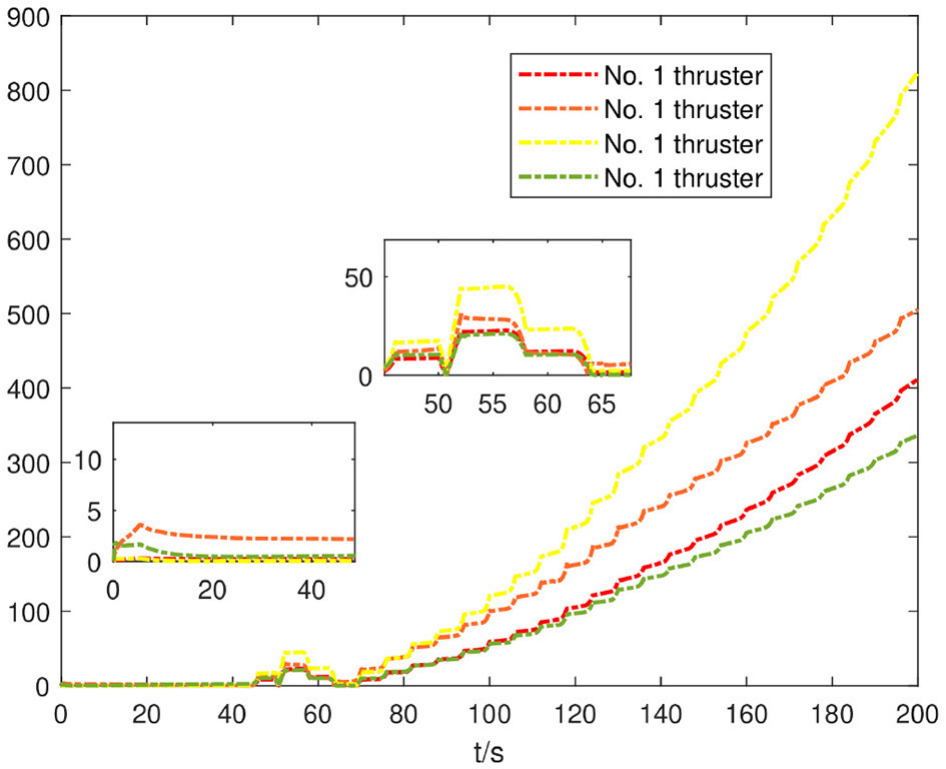
Fault adaptive curve of thruster.

As can be seen from the adaptive duration curve of Figure 8, when the system has only No. 1 thruster and No. 2 thruster failure, the adaptive rate of the PEFC controller only produces a large numerical fluctuation before 10 s to compensate the system. Due to the recovery of the stable linear tracking stage and the fluctuation failure of the adaptive duration curve, it shows that the propulsion system is still in a relatively controllable state for the desired motion attitude, but when the system is stuck and paranoid in 40 s. The adaptive curves of No. 1, No. 2, and No. 3 thrusters with faults are obviously enhanced, and the No. 4 thruster is slightly enhanced, indicating that the propulsion system needs the controller to adaptively compensate the power gap, and the desired attitude of the system can be maintained by enhancing the thruster output. It can be further proved that the fault coupling control scheme of the projection analytical separation system designed by PEFC has a better control effect when the system has complex faults.

**Figure 9:**
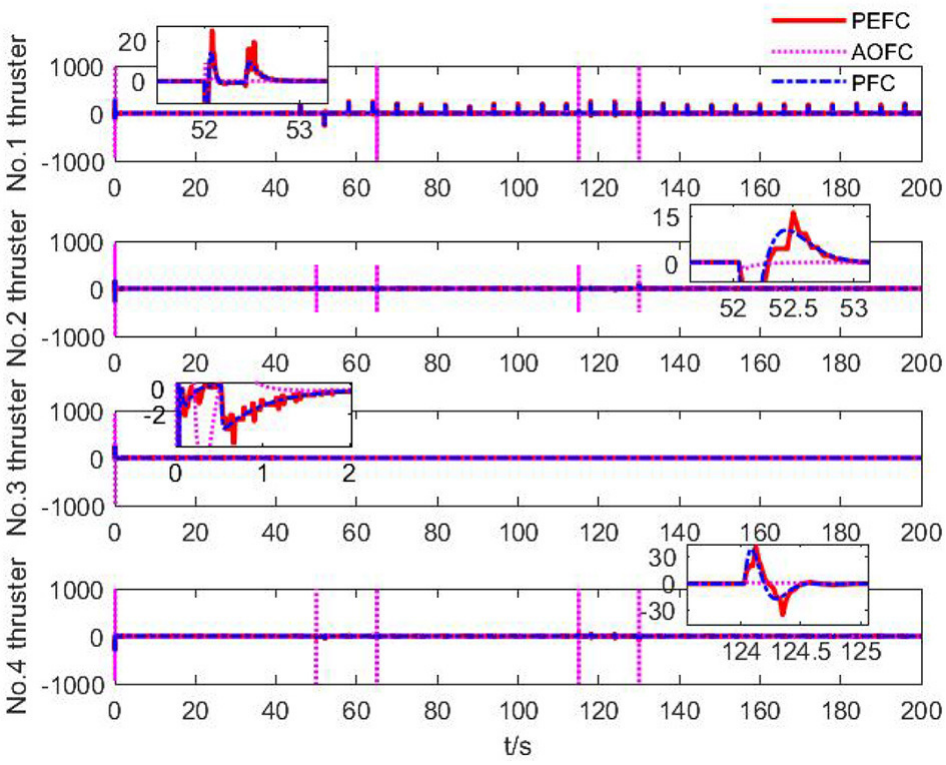
Thruster output force.

**Figure 10:**
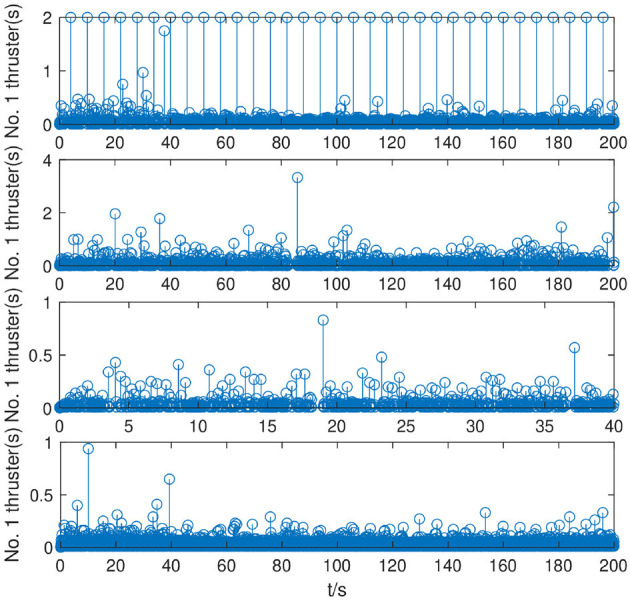
Cumulative number of thruster triggers.

**Figure 11:**
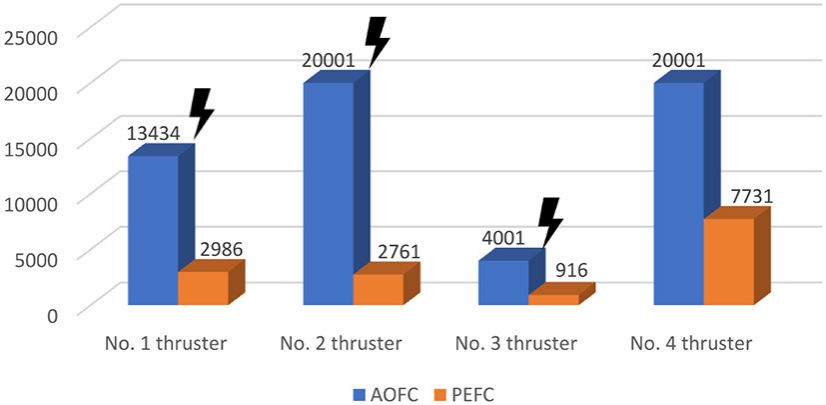
Time between thruster maneuvers.

**Figure 12:**
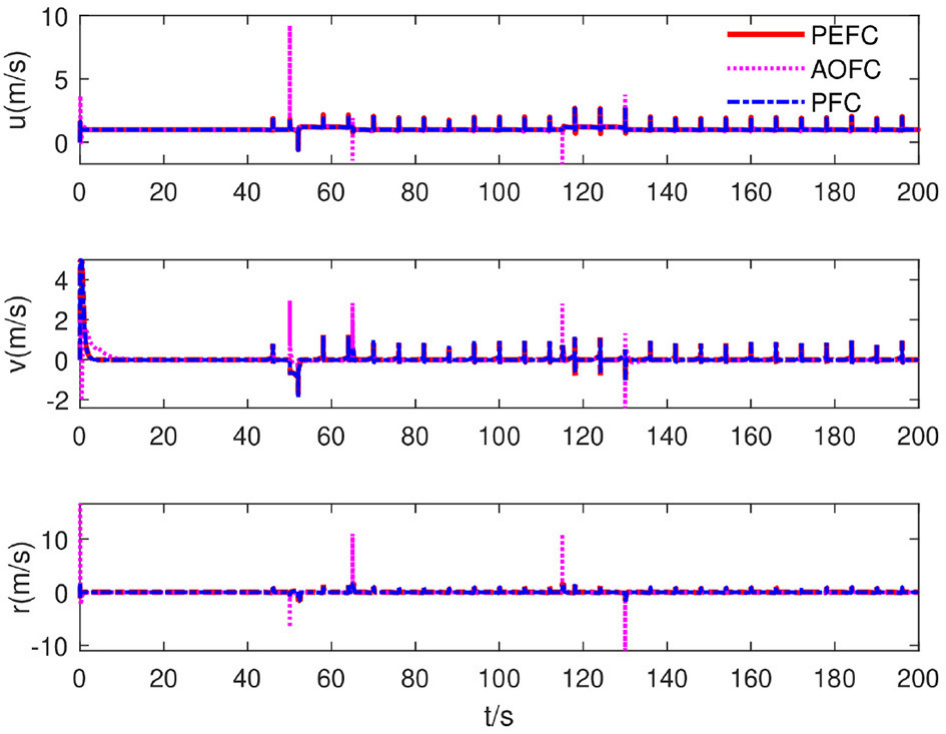
System velocity curve.

As shown in Figure 9, under the control of the PEFC controller, the thrusters of the system are more stable in the initial adjustment stage, and the thrust of the thrusters of the system can be distributed more reasonably in the medium-term trajectory tracking process. After event trigger processing, the PEFC controller is shown in Figure 10, and Table 2 effectively reduces the update adjustment frequency of the failed thruster by 75% and the thrust output of the thruster by 28.95%. As shown in Figure 11, the thruster does not need to be adjusted for a long time in the fault state while ensuring the control effect of the system, but the torque output jitter is more visible than the PFC controller. As shown in Figure 12, the PEFC control algorithm is more reasonable for the transient speed adjustment of the underwater vehicle without excessive acceleration.

**Table 2:** System simulation thrust output parameters.

	**No. 1**	**No. 2**	**No. 3**	**No. 4**	**Total**	**Saving ratio**	**Update frequency**
PEFC	14340.16	3955.78	784.06	6720.86	25800.88	71.05%	25%
AOFC	11313.94	8945.63	3286.18	12770.23	36316.00	100%	100%

## 6. Conclusion

To solve the problem of control failure due to system failure of deep-water salvage equipment under harsh sea conditions, the proposed innovative proportional logarithmic projection analysis scheme can effectively isolate the system model dynamics, amplify the fault factor state characteristics, and free the control decision from the dependence on the fault detection system and the monitoring and sensing system of the thruster to estimate the fault situation online and compensate for the precise fault. The proposed overdrive controller can configure the power output of the faulty thruster based on the power structure of the claw-holding underwater salvage robot and then use the terminal sliding mode observer and adaptive neural network to compensate for the uncertain bounded disturbance and dynamic uncertainty of the system to further improve the control accuracy. Simulation results show that the controller can use only 71.05% of the power output and 25% of the power update frequency of the conventional adaptive fault-tolerant control to complete more accurate trajectory tracking under the fault conditions of failure, interruption, jamming and poor contact, and the energy-efficient control strategy can effectively avoid further damage of the faulty thruster. In future, the influence of armored wire cable for hoisting on robot trajectory tracking control will be further studied to improve the motion control effect and accuracy of underwater salvage robot under complex underwater terrain.

## Data availability statement

The original contributions presented in the study are included in the article/supplementary material, further inquiries can be directed to the corresponding author.

## Author contributions

GG: modeling, software simulation, and writing. QZ: background research, innovation extraction, and writing review. YZ: method and format modification. WT: literature arrangement and data simulation. ZT: conceptualization and supervision. SM: paper structure adjustment and format revision. All authors contributed to the article and approved the submitted version.
